# Apparent Strength Conceals Instability in a Model for the Collapse of Historical States

**DOI:** 10.1371/journal.pone.0096523

**Published:** 2014-05-08

**Authors:** Daniel John Lawson, Neeraj Oak

**Affiliations:** 1 Heilbronn Institute, School of Mathematics, University of Bristol, Bristol, United Kingdom; 2 Bristol Centre for Complexity Sciences, University of Bristol, Bristol, United Kingdom; Georgia State University, United States of America

## Abstract

An explanation for the political processes leading to the sudden collapse of empires and states would be useful for understanding both historical and contemporary political events. We examine political disintegration across eras, cultures and geographical scale to form a simple hypothesis that can be expressed verbally yet formulated mathematically. Factions within a state make choices described by game-theory about whether to accept the political status quo, or to attempt to better their circumstances through costly rebellion. In lieu of precise data we verify our model using sensitivity analysis. We find that a small amount of dissatisfaction is typically harmless to the state, but can trigger sudden collapse when there is a sufficient buildup of political inequality. Contrary to intuition, a state is predicted to be least stable when its leadership is at the height of its political power and thus most able to exert its influence through external warfare, lavish expense or autocratic decree.

## Introduction

History has witnessed the rise and fall of countless empires, dynasties and regimes. What governs these apparently inevitable processes has been discussed across the eras [1]. Whilst growth and power seem naturally self-reinforcing, reversal into decline or collapse has impacted every state and culture not present today. Further, the fate of a nation is often tied closely to the fate of its leading class; the sudden collapse of one often leads to a similar collapse of the other [2], [3].

Within a state, influence and power are often distributed unequally. Political change is affected by many factors including visible achievements and failures, deliberate manipulation, accidents of fate and external forces. Historically, stable political states can enjoy long periods of relative growth and internal stability during which the leading class can gain a larger and larger share of the wealth and resource [4], [5]. However, the process of mounting inequality has clearly not continued forever.

Power may also change rapidly, and with great impact on the fate of apparently stable states. Whilst we do not apply our model to contemporary conflict, the clarity provided by modern media during the Arab Spring of 2011 [6], [7] illustrates the lack of simplicity in these transitions. In many cases, rebellion operated without a unified name or organisation long before any form of leadership emerged (for example, in Libya [8]), signifying a decentralised process.

We are interested in why social disorder appears rapidly from an apparently stable state. Is there a generality describing when dissident movements will receive support and when they will be ignored? Actual success of rebellion movements means acquiring military power, which is strongly dependent on technology and social structure. Those with the military power may join the rebellion if it is in their interests to do so. During peace this may seem implausible, but the toll of rebellion may rapidly change the situation.

External factors are important in determining when a state fails. Pressure from other states is clearly important and we will consider examples that involve state aggression and warfare. Many collapse events have been linked to environmental factors such as local or global climate change [9]–[13] and long term degradation of resource [14] (although there is still controversy, e.g. [15]). Such external forces clearly catalyse disorder, and are frequently a proximal cause for collapse. However, this alone does not explain why stresses are sometimes resisted and sometimes cause calamity. For example, Sassanid Persia thrived during periods in which the neighbouring Roman empire experienced agricultural decline [16]. Many collapse events occur in the absence of environmental pressure [17], with external conquest, internal conflict, or poor social, political and economic institutions playing a greater role instead. We hypothesise a dynamical process behind the social conditions that can make unrest more likely to accelerate, which will interact with external stresses.

Our model is complementary to other theories of collapse [18] by providing a game-theory or economic explanation for social assumptions. Collins [19], [20] emphasises the importance of areas at the fringe of empires, so called ‘marchlands’, which tend to be the incubators of new regimes or polities. The thirteenth century author Ibn Khaldun [21] describes a concept he calls ‘asabiya’ or ‘group feeling’ in which loyalties are nested within a state. The metaethnic frontier theory of Turchin [22] combines these hypotheses. As we predict that power equality can lead to stability, the most cohesive states should emerge from marchlands and tight-knit groups with high asabiya. Thus our economic model predicts the emergence of asabiya.

We join a recent trend of providing mathematical models for historical hypotheses (some excellent examples are [23]–[26]). Mathematical modelling cannot replace historical investigation, and general principles of civil conflict and disunity can be understood [27], [28] without the need for modelling. However, mathematics provides formal reasoning that aids generalisation and guides intuition in complex situations. A mathematical theory of collapse is a first step towards a statistically sound, data-driven comparison between hypotheses (a feat we do not attempt here). Our model is too general to be the full explanation for any specific scenario, so we consider a wide range of documented collapse events that contain qualitative similarities without claims about the critical factors in any given situation. Conceptually the model is qualitative and robustly explored by considering numerous precise instantiations, which acts as a sensitivity analysis [29] helpful for supporting (but not confirming) conclusions from qualitative data.

## Results

### A qualitative model of collapse

**Figure 1:**
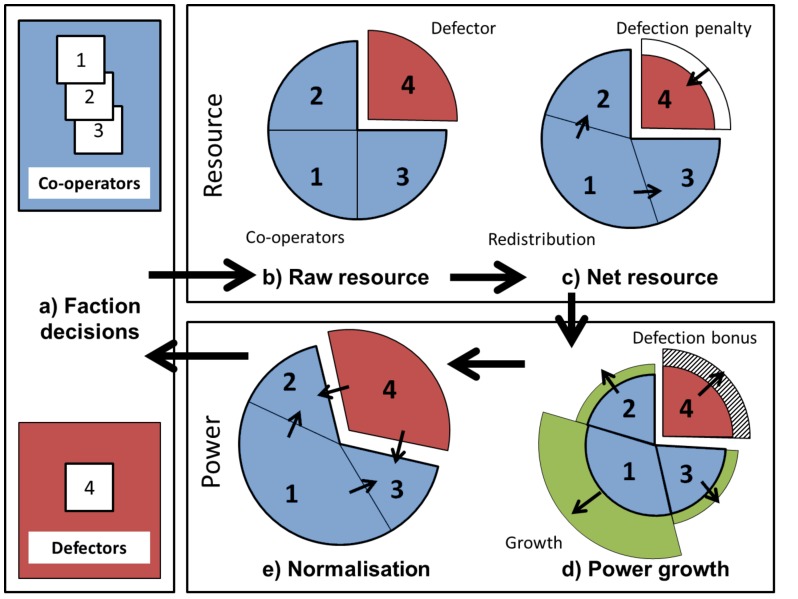
Qualitative model. Factions (a) decide whether to cooperate or defect. Then (b) raw resource is collected, which (c) is either reduced (for defectors) or redistributed according to power (for cooperators). Power grows (d) proportional to resource, with a defection bonus, and (e) is normalised so that the total power remains constant. This effectively reduces power for some and increases it for others, potentially changing their behaviour next round.

Consider a number of actors playing a repeated ‘public goods’ game, in which cooperators enter their resource into a public pool to be redistributed according to influence, which changes over time. Defectors obtain lower mean payoff but are not subject to redistribution. The game dynamics (Figure 1) draw on three vital qualitative assumptions:

1. Inequality of influence and hence resource will increase (on average) over time when actors cooperate.
2. Defecting produces an overall cost, reducing resource for the defector and reducing the public goods for the cooperators.
3. Defecting decreases the future influence inequality.

Cooperating means obeying the rules of a political system designed to prevent costly conflict between the actors. Within the system, political influence tends to accumulate with those that have the most resource, leading to increasing inequality. By ‘defecting’ from the political system, actors pay a cost but increase their political standing.

To apply this model to historical states, we interpret:

- Factions as actors;
- The state as a set of factions obeying a rule of redistribution;
- Cooperation as agreeing to the redistribution;
- Defection as refusing the redistribution, which has a cost but increases political standing;
- Political power as changing relatively smoothly and as determining resource redistribution.

We investigate how these assumptions lead to coordinated activity such that actors cooperate for long periods, and defect en-masse. This dynamic may provide an explanation for the long-term difficulties experienced by many co-operation systems, including the disintegration of powerful nation-states and empires over the course of history.

### Qualitative trends in history

In this section we outline some examples of qualitative features that are consistent with our model. The examples are necessarily selective and are chosen to demonstrate a) that our qualitative assumptions are widespread, and b) that there is value to an intrinsic understanding of state collapse.

#### Key prediction: Collapse is ubiquitous and occurs at peak power

The phenomenon of collapse has occurred across diverse world cultures throughout history, and has affected polities of all sizes. Here we describe cases where a strong state, which was able to exert significant power, has experienced collapse or unrest. Such states have strong leadership relative to their subjects, evidenced by a) the successful imposition of will, or b) the ability to expend resource in prolonged offensive wars, extensive building programs, etc. These examples contrast slow decline in which the ultimate collapse does not occur at a period of strength. In the following examples and many more, collapse or unrest followed displays of strength relative to contemporary states and/or the same state at different times.

The 5th century BC saw the peak of the Achaemenid Persian Empire, which was then the largest empire in history. The early reign of the emperor Xerxes, following that of his father Darius, was beset by internal rebellion in Babylonia and Egypt. Darius' reign involved extensive military activity, and while Xerxes was eventually able to return to the offensive, he had to start his reign regaining dominion over the territory he inherited [30].

Alexander the Great's empire is a prime example of fragmentation of a powerful state at its peak in 323BC. The division can be thought of as a collapse, as it resulted in violent upheaval [31]. The successor states or Diadochi engaged in extensive warfare over the following decades, and many subdivided further [32].

The Roman Empire underwent several cycles of expansion and instability in the first and second centuries AD. For example, the reign of the Roman emperor Domitian was lambasted by writers of his time as being tyrannical and autocratic [33]. Contemporary detractors claimed that he ignored tradition, executed senators who opposed him and openly asserted primacy over the senate [34]. After his assassination, the new emperor Nerva had a short and impotent tenure, due to revolts by the military. The indulgence and autocracy of the Roman emperor Commodus [35] is often described as the beginning of the decline of the Roman empire [4], leading to a protracted civil war. Yet the Nerva-Antonine dynasty that preceded Commodus was amongst the most successful periods in Roman history. The remnants of the Roman empire, centred on Byzantium, exhibited periodic instability well into the Middle Ages [36].

Beginning in the 6th century after the adoption of Islam, Arab armies expanded their domain westwards towards Morocco, east into Persia and as far north as France. The empire collapsed in the mid-8th century, and the successor states suffered further internal conflict and dissolution in the following centuries [3].

Contemporaneous to European medieval civilisation, the Khmer civilisation of Indochina achieved regional hegemony and its capital Angkor may have been the largest city in the world [37]. It experienced multiple damaging episodes of civil war, notably prior to the accession of Suryavarman I [38]. Angkor provides an excellent example of a state whose power rested on appropriation from vassals; indeed, much of Jayavarman II's power depended on a rice surplus [39]. Its ultimate collapse is associated with climate [12],

Finally, the Mongol empire was the largest contiguous land empire in history. In the wake of their initial conquests the empire divided, at times violently, into smaller, culturally heterogeneous polities [2].

#### Prediction: Cascading civil unrest

It is not clear a-priori whether states should end rapidly, slowly, or require external influences to fail. A brief examination of history leads to the conclusion that civil unrest can very quickly accelerate, leading to at least the potential for rapid state collapse. In history and in our model, uncoordinated defections often produce their own momentum. As more factions choose to leave a state, the perceived legitimacy of that state is eroded encouraging further defections.

This is seen in many of the examples discussed, but we draw attention to the Seleucids and the Byzantines. The furthest vassals of the 1st century BC Seleucid empire seceded early, leading to a geographical wave of defections [31], [32]. In the middle ages, the Byzantine emperors were frequently beset by a single rebellion or usurpation that preceded rebellions around the empire [40]–[43].

#### Assumption: Inequality before unrest

Our model assumes that perceived inequality can influence the decision to defect. This is backed up by unrest frequently following both centralisation and increasing inequality. Once again, examples can be found across documented history.

From the 14th century BC, an excellent example of unrest stemming from centralised power is the reign of Amenhotep IV (Akhenaton) of Egypt. He introduced massive centralisation in the form of a new religion. The strength of his regime is clear from the foundation of a new capital and decoration of the empire with sun temples and other paraphernalia. His reign was followed by unrest and internal disorganisation [44].

Many examples of inequality leading to unrest can be taken from the late Roman republic in the first century BC. The Social (or Marsic) War [5], as well as three Servile Wars were fought by Rome during or immediately after periods of expansion abroad. The stated grievance in these cases was explicitly inequality; the slaves wished to be elevated from their abject position in society, whilst the Socii demanded an end to their second-class status within the Republic. Caesar's civil war [45] also took place immediately after a period of offensive foreign campaigns, notably the annexation of Gaul. Caesar explicitly states that inequitable distribution of wealth and power was a cause.

A small scale but important example was the reign of Richard I of England in the 12th–13th century AD, leading to the signing of the Magna Carta. To fund conflict in France and the Levant during the reign of Richard I, the crown made increasing demands on vassals. Following this, John I was forced to make concessions to the nobility [46]. This can be thought of as an example of factions resisting an attempt to appropriate resource and power.

As a final example, the 1905 [47] and February 1917 [48] revolutions in Russia are often attributed to the inequitable distribution of wealth and power between the ruling classes and the majority of the population [49] – the term ‘autocrat’ was even part of the official title of the Tsars.

#### Assumption: All else being equal, power will agglomerate

Our model makes the assumption that power tends to accumulate over time. Although there are counter-examples, the tendency towards conglomeration is relatively ubiquitous. Specific examples include the concentration of land holdings into the latifundia of ancient Rome [4], the extensive provincial landholdings of the late Sassanid empire [50], and the distinction between land-holding nobility and the rest of society in Feudal Europe [51]. Mercantile quasi-states such as the Hanseatic League [52], Italian merchant republics [53] and European colonial enterprises [54] exhibit this behaviour, amongst many others.

#### Assumption: Defection can be pragmatic

We have assumed some degree of rationality about the choice to revolt. Whilst we are very careful not to assume that people are rational, we must assume that there is some link between the resource expected from defecting, and the choice to defect.

As an extreme but clear cut and common example, one instigator of internal conflict is the inability of a faction to hold political power or influence by peaceful means. This occurs when a faction perceives that it receives an inadequate degree of power, or that it's power is being eroded. The perception of inequity may encourage the use of violence in order to redress the apparent imbalance. Excellent examples of this appear in the late Republican period of Rome [55]; of particular note is the career and attempted rebellion of Catiline [56]. The repeated, and often unsuccessful, peasant revolts of medieval Europe also exhibit many of these features [57].

### Basic model: State formation and collapse

The model takes the form of an iterated multiplayer game. Consider 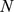 autonomous factions (i.e. actors), who may be individuals or groups with similar enough motivations to act coherently, competing via the model in Figure 1 (defined precisely in Methods). Each faction starts with an equal amount of raw resource and chooses to defect or cooperate. Actions are chosen in order to obtain the highest net predicted resource by predicting that other factions do not change their decisions (the assumption of pragmatism). Net resource depends on all factions' actions and power, treated as a zero-sum game (which is an oversimplification [58]). Power is then updated based on the resources obtained and the decisions made. Power increases most when a large amount of resource is obtained (assumption of conglomeration) or when defecting (an implicit assumption that there can be no ultimate winner). The process is then repeated using the new distribution of power and decisions.

**Figure 2:**
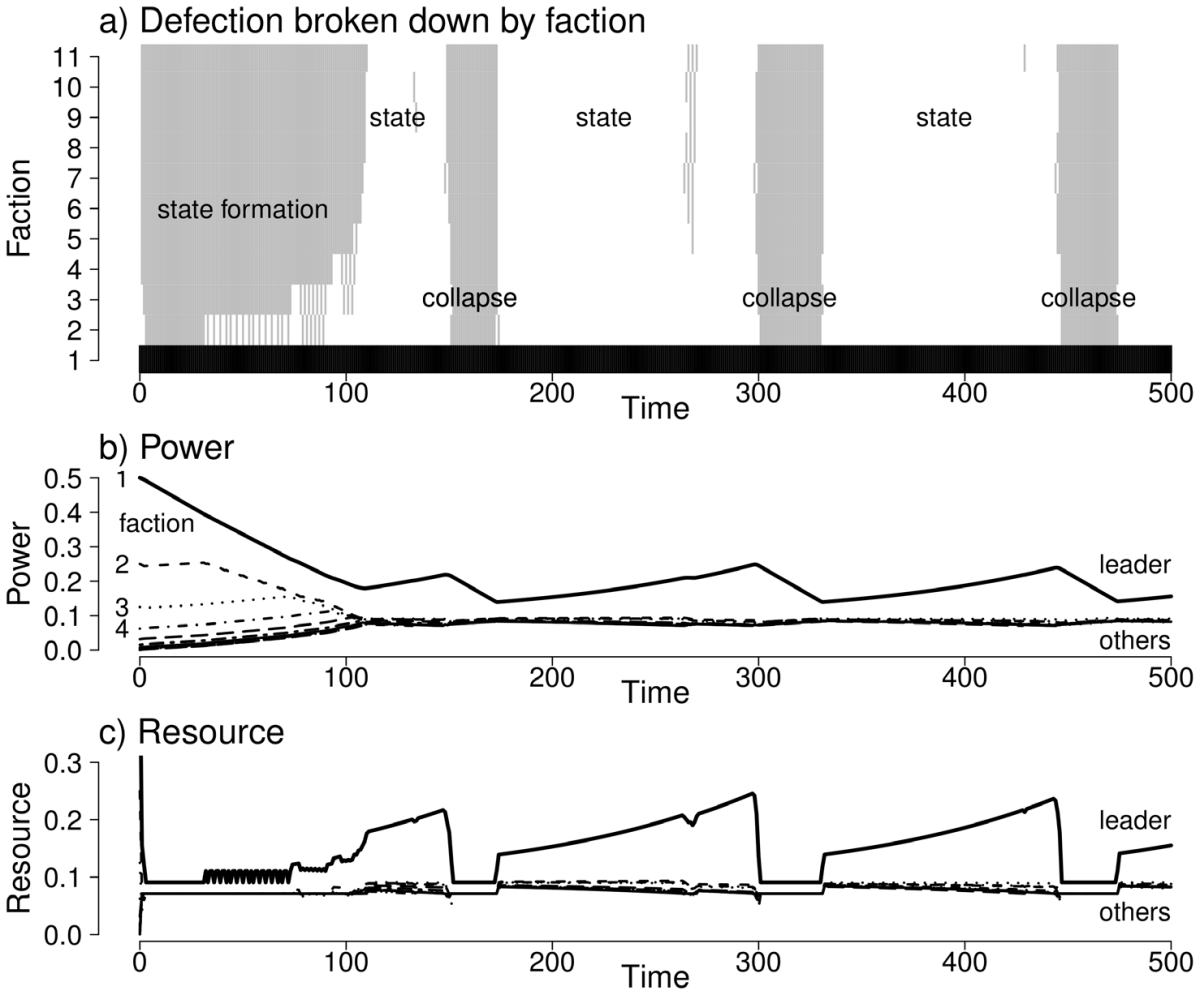
Characteristic behaviour of our model. a) Defection behaviour with state formation and collapse. Defection is shown in grey, cooperation in white, and the leading faction in black (which always cooperates). b) The power of factions over time. c) The resource of factions over time. The power and resource of the non-leader factions converge, with the result that periodic coordination and defection periods occur. (Parameters: 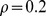, 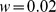, 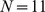 and 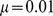.)

The model produces periods of widespread cooperation and collapse, as shown in Figure 2. During state formation, the power of non-leader factions equalises around the point at which cooperation becomes viable. Once cooperation is established, defection occasionally occurs in isolation as redistributed resource falls below a threshold. Infrequently, enough factions are sufficiently weak so that the defection of another faction changes their own best choice, leading to a defection cascade. Once the political landscape has equalised sufficiently, a corresponding cooperation cascade occurs nearly as rapidly. In this simple model, cooperation and defection periods occur with a predictable timescale leading to ‘spontaneous’ periodic behaviour.

**Figure 3:**
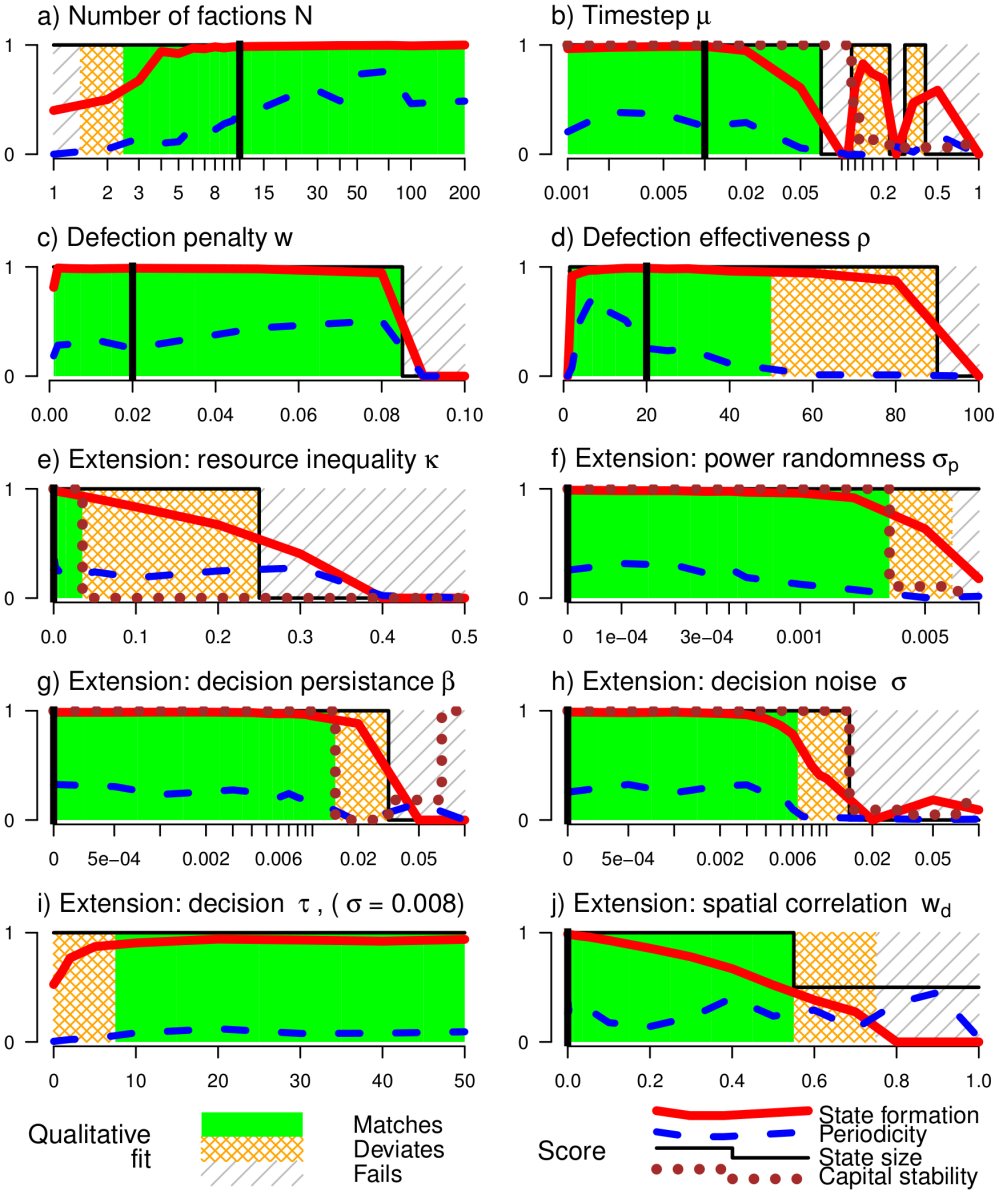
Effect of parameters/model extensions on the qualitative dynamics. The plots are shaded to show whether model qualitatively behaves as Figure 2. The model either matches (solid), deviates (dense hatching) or fails (thin shading). The qualitative fit is based on quantitative scores (see Methods). Firstly, ‘State formation’ (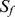) is high when states are large and collapse rapidly to few factions. Secondly, ‘Periodicity’ (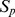) is high if there is periodic predictability to decisions. Thirdly, ‘State size’ (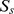) is high if state formation and collapse affect all factions. Finally, ‘Capital stability’ (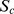) is high if the leading faction does not change from the initial leader (relevant only for plots e–h). The qualitative model is matched if 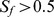, 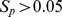, 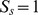 and 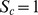. It deviates if 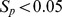, 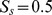 or 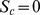. Otherwise the qualitative model fails. Also shown (where possible) is the parameter value from Figure 2 (vertical line).

How reliable is the model? It has four parameters: the number of factions 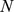, time discretisation 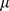, defection resource penalty 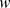, and defection power gain 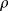. Figure 3a-d highlights the parameter regions for which the model behaves as Figure 2. The figure shows a number of qualitative traits, which are summarised into three categories. For many parameters there is a ‘match’ with Figure 2, meaning that large states form, that collapse occurs both rapidly and completely, the leading faction remains the leader forever, and the state survives for a predictable period of time. ‘Deviation’ relaxes some of these constraints: states must still collapse quickly and completely, but are not required to include all factions; the leading faction is allowed to change, and states may be of variable duration. ‘Failing’ to qualitatively fit means that collapse is never complete, or no state forms. These are defined precisely in the methods.

Although 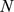 has many important effects (Section S1 in File S1), all values of 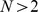 match the qualitative model. Similarly, all theoretically valid values of the defection penalty 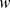 also match (Section S1.3 in File S1). If the timestep 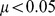 is small it acts as a timescale, but large 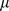 or rebellion effectiveness 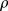 prevents periodic behaviour due to ‘intrinsic’ noise (see below). We will also see later that small changes to our model can lead to unpredictable periods of cooperation, but that the collapse of ‘strong’ states is relatively ubiquitous.

#### Features of the basic model

We can take the continuous time limit of our model, which removes intrinsic noise due to discretisation. We can also take the continuous faction limit, which leads to a Partial Differential Equation model. These models (Section S1 in File S1) are not readily solvable but do allow us to understand why our model behaves as it does.

During cooperation, the power of each faction departs exponentially from the baseline. Defection cascades occur because:

1. A defection always makes cooperators worse off;
2. Later defections have a greater impact than early defections, making a cascade more likely as more factions defect;
3. Failed defection cascades erode the power of weaker non-defecting factions most, helping future cascades to succeed.

The dynamics follow 4 distinct phases that repeat in a cycle:

1. Cooperation: Power becomes concentrated in the leading factions. Weaker factions may defect in an uncoordinated manner.
2. Collapse: Defectors coordinate into a cascade when the cumulative power distribution is everywhere above a threshold.
3. Defection: Defection continues until power becomes sufficiently diffuse to permit cooperation. The strongest factions may cooperate first in failed state formation attempts.
4. Recovery: A cooperation cascade occurs in much the same way as the defection cascade, when the cumulative power distribution is everywhere below a (complex) threshold.

Additionally, we obtain a bound on the duration of cooperation and defection periods by allowing all non-leading factions to behave identically. In this case we can obtain closed-form expressions for the duration of cooperation and defection phases. The initial conditions can be very important in determining how close the bound is, from which we conjecture that this model has no general analytic solution, although bounds can be found and special cases solved.

### Extensions: A model sensitivity analysis

When performing parameter inference using quantitative data, a minimum requirement is to assess how robustly the parameters are inferred via a sensitivity analysis [29]. Here we are instead trying to infer that some qualitative features were created by a general class of model. We attempt to understand the qualitative model space using a model-level sensitivity analysis, i.e. by making changes to the model and observing how the predictions vary.

#### Unequal resource distribution

Resource is distributed unevenly in practice, which we model by replacing the raw resource 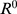 with 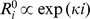 for faction 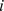. 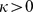 means that initially powerful factions have less resource. Figure 3e shows that a resource-weak leader can either persist or be usurped. Periodicity and collapse events persist, and further, Section S2.1 and Figure S1 in File S1 shows that a resource-weak leader results in reduced average conflict.

#### Uncertain outcomes

The political power process is contingent on events outside of complete control of faction leaders. We model this by adding noise (normal, with mean 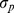; see Section S2.1 in File S1) to the obtained power change before normalisation. Figure 3f shows that small levels of noise do not effect the qualitative behaviour. Moderate levels lead to a leader turnover and uncertain state lifetimes, whilst high levels prevent both coordination of both state formation and collapse (Figure S2 in File S1).

#### Biased decision making and random choices

People are not naive resource optimisers. Decisions may be biased, hard to change, poorly calculated, or made with respect to longer term goals and otherwise unobserved features.

Complex decisions can be allowed for by introducing a *random function* 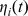 for the decision threshold of each faction. We include ‘persistence’ via a bias 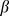 towards the previous action, and random fluctuations in whether to favour defection or cooperation via a *Gaussian Process* [59] (Section S2.3 in File S1). This is determined by two parameters: the magnitude of the fluctuations 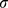 and their correlation over time 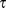. To interpret 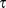, factions are effectively making ‘new random decisions’ every 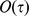 time units. If 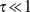, then decisions appear ‘noisy’, and if 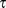 is very large, each faction will appear (randomly) biased.

Figure 3g and Figure S3 in File S1 show that a range of 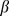 has no qualitative effect, whilst moderate values lead to unpredictable leader turnover, make both the magnitude of a collapse event and the duration of a stable period uncertain. Figure 3h-i demonstrate that small to moderate levels of noise (Figure S4 in File S1) don't effect the dynamics, and further, when decisions are more correlated in time (Figure S5 in File S1) then state formation is more stable, even in the presence of high decision noise. This happens because power has time to equilibrate around the random choice of decision boundary; i.e. factions adapt to the stubbornness of other factions, and adjust their own bargaining positions accordingly.

#### Spatial structure

Some political scenarios are best described with a spatial model. For example, factions may be local leaders of villages, or semi-autonomous regions of a larger state. We replace 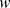 by 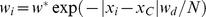 (Section S2.4 in File S1), i.e. both the resource penalty for defection, and the political gains from doing so, decay with distance from the capital (leading faction). The average 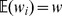, i.e. is unchanged, and distance is calculated on a ring (so factions 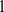 and 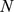 are neighbours).

The spatial model (Figure 3j) allows for a variety of different scenarios. The state grows from the capital (Figure S6 in File S1) and collapses as in the non-spatial model. Collapse may be from the outside in, or the inside out. There may be a well defined maximum spatial extent (hatched parameter region of Figure 3j).

#### Modified intrinsic noise

We chose to define Model 1 as an iterated game, which has consequences for the way that noise enters the system. Although the basic model is deterministic, the discretisation of time can produce ‘chaotic’ dynamics (e.g. large 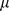 in Figure 3b) as small variations in the value of political power have large effects. Is this ‘intrinsic noise’ important for the dynamics?

To address this issue we constructed a modified version of the model in which only a single faction makes a decision at a time (using the Gillespie Algorithm [60]) with an average timestep of 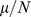 (Section S2.5 in File S1). Figure S7 in File S1 compares this model with the basic model and shows that there is no qualitative change. Additionally, the continuous time version of the model (Section S1 in File S1) matches the qualitative data. We view these issues as ‘modelling degrees of freedom’ and only consider model behaviours that are present for all choices.

#### Non-uniform defection penalty

If the penalty for defection decreases with the number of defectors, both defection during cooperation and cooperation during defection are harder. This makes the phenomenon of collapse more likely to occur, as we show numerically (Section S2.6 and Figure S8 in File S1). Leader replacement is also easier under this model, if there is at least some noise.

#### Non-linear relationship between power and resource

Power and resource are simply related in our model. However, we find that a family of non-linear functions do not effect the qualitative dynamics (Section S2.7 in File S1). Since we can map resource levels to a decision boundary in the power distribution, we conjecture that most ‘reasonable’ increasing functions will demonstrate collapse.

### Game theory perspective

We have not permitted factions to consider politics when making decisions. Does our model still describe collapse if longer term strategies can be employed? A little game-theory analysis shows that the main phenomena persist, and further, that the game has interesting behaviours of its own.

The resource payoff in our model takes the form of a simple iterated (multiplayer) game. Consider the case where there are two factions, 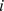 and 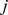 (with 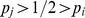 and 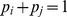) having the payoff structure given in Table 1.

**Table 1:** Payoff structure for the two player version of the game, when *p_i_<p_j_*.

Payoff for 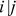	*j* defects		*j* cooperates	
*i* defects	1/2—*w*	1/2—*w*	1/2—*w*	1/2
*i* cooperates	1/2	1/2—*w*	*p_i_*	*p_j_*

In this case *j* should always cooperate.

Until now we have assumed that factions are simple resource maximisers. Whatever 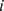 does, 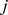 always obtains more resource from cooperation. Since 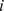 knows this it should cooperate only if 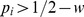. This is how we derived the behaviour for our factions in Model 1. In this circumstance 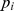 increases when 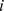 defects, and decreases when it cooperates. In Model 1a where decisions are continuous, 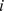 changes action around the decision boundary and obtains payoff 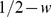 whether cooperating or defecting.

However, 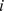 could attempt to maximise its payoff over all time. There is no reason for 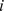 not to defect for the power benefit, since only the defection payoff is obtained on average. If 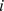 were to defect until power is equalised, then it would enjoy a long period of high resource until power became uneven again. The payoff from becoming the leading faction is even higher.

Should 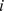 agree to cooperate if 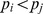
? If not, and 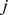 uses the same reasoning, then both players will on average get the ‘greedy’ payoff 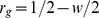 (as they have to defect half of the time). If either were willing to take the lower cooperation payoff they would get more over time. Proposition 1 shows that there is a strategy which maximises the long-term resource payoff:

#### Proposition 1

*For Model 1a (the continuous time model) with two players, there exists a defection strategy defined by a power lower bound* 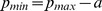 *for a given upper bound* 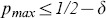 *with* 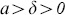, *which when both players use it the resource obtained is* 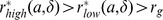 *for the stronger and weaker players respectively. (For proof, see* *Methods*).

The existence of this longer term strategy leads to an interesting extension. We now allow 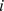 and 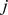 to use two potential strategies in a long-term meta-game, which both dominate the short term strategies. Passive players use the strategy from Proposition 1, and aggressive players cooperate only as the dominant faction. We consider the payoff averaged over many cycles, assuming that during each state formation the player with the initially higher power is chosen randomly. Therefore a passive player always ends up with lower power than an aggressive player, two aggressive players obtain the ‘greedy’ payoff 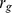, and two passive players share leadership over time. The average payoff matrix is given in Table 2.

**Table 2:** Long term payoff for strategies taking into account future payoff in the two player version of the game, averaged over many iterations.

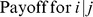	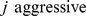		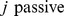	
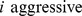	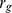	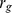	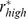	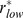
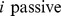	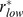	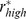	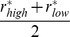	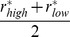

“aggressive” and “passive” are defined in the main text, as are the payoffs 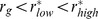.

As 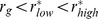, this is a form of the prisoners' dilemma. We note an analogy of the ‘passive’ strategy with democratic parties sharing power over time, as opposed to corrupt or autocratic systems in which this is impossible.

The case of three or more factions can be understood analogously. Optimally, weaker factions will act together to remove power from the leader, and such coordination follows naturally from the ‘passive’ strategy. Consider that factions cannot solve for the optimal thresholds 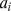 but can choose them empirically. Cooperation first occurs when all factions have equal power (to within 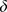). Each defection now occurs at the factions' chosen threshold. As in the simple model, defections reduce cooperators' resource leading to defection cascades. Aggressive factions still do not cooperate unless they are the leader and will not take part in the state. We therefore conjecture that any number of short-term resource maximisers, and/or ‘passive’ long-term strategists, can form states that experience dramatic collapse events.

## Discussion

Why do states vary in their susceptibility to stresses, and is state failure likely to occur in the absence of external pressure? Although inequality appears to increase over time under the status quo, clearly this status quo has not continued forever. Our main result is that inequality redress will typically take place in large scale events and can include complete state collapse.

The model should be interpreted as representing a very simplistic form of political state. Factions within the state produce higher resource than those defecting. This could be because the machinery of the state makes them more efficient, or because there is a cost to not being a part of a state (in terms of lost trading opportunities, etc). It does not imply a complex bureaucracy and is therefore appropriate for many groups of people, including states, chiefdoms [61] and potentially more modern concepts such as democratic parties and corporations (although we do not explore these links here).

Cooperators in our model do not pay a price for punishing defectors (except losing their redistributed resource). Because we are interested in collapse, not state formation, we chose to make states as robust as possible. To this end, factions that choose to cooperate receive only indirect penalties when others defect. Much work (e.g. [62], [63]) has gone into understanding how cooperation can arise even if there is initially a marginal penalty to cooperation when it is rare. Our ‘non-uniform defection penalty’ model still supports state formation and is closer to this scenario, as the costs vary smoothly as the state grows. However, there is always a marginal benefit to cooperation.

A vitally important point is that our model (like all models of complex systems) is a gross simplification of reality. External effects including climate variability, long term climate change, conflict with other states, and contingent processes such as a lack of heirs or an objectively poor ruler are all extremely important in determining the time and severity of real political calamities. Although we find that collapse events will occur in the absence of external pressure, our claims do not extend to their duration, nature or proximal cause. Our interpretation of state collapse means significant internal unrest but further assumptions are required to predict the failure of important institutions, whose survival depends on the severity and duration of the unrest as well as the support of the *de-facto* leadership.

The difficult task of a detailed empirical evaluation of this and related dynamic approaches has been left to future work. Although our model describes an interesting phenomena for long-term social interactions, it is hard to prove historical relevancy. Compelling validation would require collating the inherently patchy and qualitative evidence into quantitative data from a wide and approximately unbiased range of sources, and interpreting it in a manner that can be agreed on by all researchers. This is a bar is that difficult to reach in social science, although recent efforts towards historical quantification [64] begin the first steps of this process and [65] makes a compelling attempt to validate models with data. Without widespread exposure to data, all historical models including our own and those we have cited have been selectively and qualitatively validated and can only be disproven by qualitative comparisons.

We have made a significant effort to legitimatise the use of a utility function for faction behaviour, by incorporating random time varying functions into the decision process. Many factors influence decision making, from alternative goals to incomplete knowledge, without a need to address whether the choices are rational. We make the qualitative assumption that the resource difference between defection and cooperation will correlate with the choice a faction makes.

Although collapse seems inevitable in our model, real states may use unmodelled options. Deliberately limiting state power to maintain an equitable resource distribution would work in our framework, and is attempted by many modern states. Conflict resolution between the state and disgruntled factions might also be possible. However, states that have not experienced a defection leading to widespread disorder are likely to underestimate the danger posed by resolution failure (an example of ‘availability bias’ [66]). This can lead to an unexpected defection cascade that will rapidly become impossible to control. Therefore under our assumptions preventing the buildup of political inequality is a better solution for state stability than responding to specific grievances.

Conflict is considered as a reduction of resource in our model and so force is used only indirectly (e.g. imposing sanctions). This may seem unrealistic when many rebellions are put down violently. Internal conflict is difficult to model because factions are fluid concepts and the elimination of defecting factions could cause the remaining factions to splinter. Our assumptions hold best for political systems that dissuade escalating warfare. Examples are coalitions of city states such as in Ancient Greece, medieval feudal lords who share genealogies and culture, and parties within democratic systems who can slow the implementation of policies of the ruling party at a productivity cost to the state.

We are cautious about the conclusions that can be drawn from our model, but believe it is still valuable for three reasons. Firstly, we provide the intuition that near-stability need not be the default state of affairs despite long stable periods being common throughout human history. This is the most general explanation to date of the apparent contradiction that history's most powerful empires have ultimately and often rapidly failed, frequently despite military and economic advantages. Secondly, we have examined this process in a logically rigorous mathematical framework. The verbal model makes intuitive assumptions, and whilst our predictions also make intuitive sense *post-hoc* the mathematical modelling is needed to establish that dissatisfaction does not dissipate in small scale events. Finally, we have explored how this model compares to history and found qualitative evidence that the power dynamics we describe for inequality have been important.

## Methods

### Mathematical model

The decision to defect 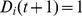 if the predicted relative resource obtained from defection 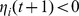, i.e. is negative. Cooperation, i.e. 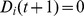 occurs otherwise. The predicted relative resource is given by
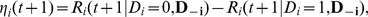
(1)and 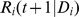 is the predicted resource obtained by action 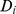 (assuming all other factions do not change). Cooperators pool and redistribute resource, 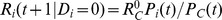, where 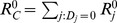 and 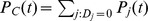. Defectors retain resource with a penalty, 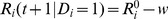. Power changes according to
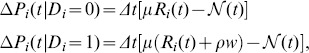
(2)where 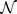 is a normalising constant to ensure 
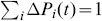
. In the basic model 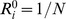. Power is initialised by giving each faction half the power of the previous one.

### Qualitative indicators

Figure 3 quantified the match of our model to the qualitative data by use of four indicators. These crude measures match our intuitive understanding of our model. These measures take as input the second half of a long (4000 time step) run to ensure that we are observing the long-run behaviour.

1. ‘State formation’ (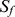) measures the proportion of time the number of conforming or defecting factions is very high or very low. A condition for a ‘state’ in our model is that it retains a high proportion of the factions. Specifically: we tabulate the number of timesteps the each number of factions 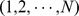 defects. These are split into quartiles 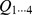 (accounting for non-uniform bins). The score 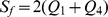 measures the proportion of time the state either exists or does not exist, discounting transition times.
2. ‘Periodicity’ (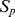), measured by how predictable conforming decisions are in 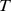 timesteps. Specifically, we form the probability of a match 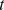 timesteps apart, 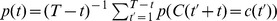 for the number of conforming factions 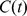. We find the time difference 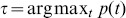 for which 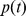 is maximal (excluding the first mode at 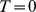), and then report the height of the probability peak as the score 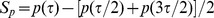 (comparing the height of the largest peak to the height of the ‘troughs’ each side of it). This produces similar estimates of 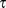 to the (more standard) autocorrelation function but in practice resulted in slightly smoother estimates of 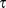 and 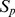.
3. ‘State size’ (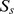), scored as 1 if all factions have simultaneously cooperated and simultaneously defected, 0.5 if they all simultaneously defected, and 0 otherwise. This allows us to detect collapse either in full-sized, or smaller states.
4. ‘Capital stability’ (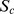) is 1 only if the leading faction does not change from the initially most powerful faction, and 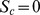 if it has done at any point in the history.

We convert from scores into a qualitative match using the requirements from Table 3.

**Table 3:** Conversion between quantitative scores and qualitative indicators of model fit.

Score	Match	Deviate	Fail
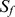	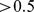	NA	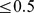
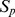	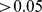	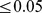	NA
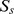	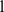	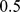	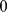
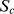	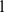	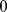	NA

Scores are ‘State formation’ (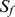), ‘Periodicity’ (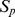), ‘State size’ (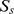), and ‘Capital stability’ (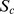) defined precisely in Methods. “Match” and “Deviate” are both acceptable fits to the historic data.

For a simulation to be classified as a ‘match’ all individual scores must match. To be classified as ‘deviation’ one or more individuals scores must deviate and all others match. If any scores fail then the model is classified as ‘fail’. Notice that deviation implies either low periodicity, incomplete states, or changing capital location, all of which are historically plausible.

#### Proof of Proposition 1

Power during cooperation follows:
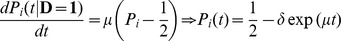
starting at 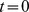. The time taken to reach 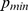 is 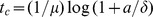. The resource obtained per unit time in the cooperation state is 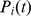. Therefore the total resource obtained is:
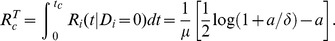
During the defection phase, resource is obtained at rate 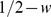 and power accrued at rate 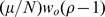, so the defection duration 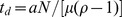 and therefore 
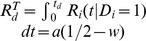
. The average rate of resource acquisition is
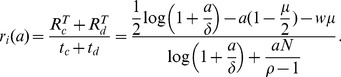
(3)This can be solved for 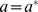 giving a maximum resource 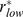 when the resource rate 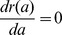. Although there is no explicit form, the maxima exists at positive 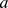 and is non-trivial (i.e. not a boundary). During this time, the leading faction obtains a higher payoff during the cooperation phase 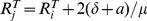, with a higher average rate 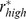.

## Supporting Information

File S1 **Mathematical details.** This can be read as an extended methods section. All of the supporting information including Sections S1–2 and Figures S1–7 are contained within. Section S1 derives the ordinary and partial differential equation models (Models 1a and 1b). These allow calculation of the dynamics of power in cooperation and defection phases (Section S1.1). From this we can obtain defection thresholds for each faction (Section S1.2). Bounds for the duration of cooperation and defection (Section S1.3) are calculated by assuming that all non-leading factions have the same power. Some equivalent reformulations (Section S1.4) are provided. Section S2 describes each of the model extensions described in Section, with Figures S1–7 showing samples from each of these models in the same format as Figure 2a.
(PDF)
