# Supplementary material for: Apparent Strength Conceals Instability in a Model for the Collapse of Historical States
Source: PLoS One. 2014 May 8;9(5):e96523. doi: 10.1371/journal.pone.0096523 (PMC4014536; doi:10.1371/journal.pone.0096523)
Supplement: File S1 — Mathematical details. This can be read as an extended methods section. All of the supporting information including Sections S1–2 and Figures S1–7 are contained within. Section S1 derives the ordinary and partial differential equation models (Models 1a and 1b). These allow calculation of the dynamics of power in cooperation and defection phases (Section S1.1). From this we can obtain defection thresholds for each faction (Section S1.2). Bounds for the duration of cooperation and defection (Section S1.3) are calculated by assuming that all non-leading factions have the same power. Some equivalent reformulations (Section S1.4) are provided. Section S2 describes each of the model extensions described in Section, with Figures S1–7 showing samples from each of these models in the same format as Figure 2a. (PDF) [file pone.0096523.s001.pdf]

Supporting Information for 'Apparent strength conceals instability in a model for the collapse of historical states'

Daniel John Lawson*and Neeraj Oak†

April 15, 2014

Contents

S1 Mathematical frameworks for the basic model **2**
S1.1 Behaviour when all factions cooperate 3
S1.2 Defection cascades 4
S1.3 Duration of epochs in the cycle 5
S1.4 Informative reformulations 6

S2 Model Extensions **7**
S2.1 Unequal resource distribution 7
S2.2 Uncertain outcomes 7
S2.3 Biased decision making and random choices 9
S2.4 Spatial structure 11
S2.5 Modified intrinsic noise 15
S2.6 Non-uniform defection penalty 16
S2.7 Non-linear relationship between power and resource 18

List of Figures

S1 Unequal resource distribution 8
S2 Uncertain outcomes 9
S3 Biased decision making 10
S4 Random choices with uncorrelated noise 12
S5 Random choices with correlated noise 13
S6 Spatial structure 14
S7 Modified intrinsic noise 16
S8 Non-uniform defection penalty 17

*Heilbronn Institute for Mathematical Research, University of Bristol, Bristol, BS8 1TW, UK
†Bristol Centre for Complexity Sciences, University of Bristol, Bristol, BS8 1TW, UK

S1 Mathematical frameworks for the basic model

Model 1a removes intrinsic noise (see Section S2.5) by taking the continuous-time limit. Instead of factions being offered a choice to defect according to a schedule, time proceeds until the next change in decision occurs. Multiple decision changes may follow instantaneously if the choice of one faction affects the choice of another. In this way we can understand 'defection cascades' and 'cooperation cascades' in which a large proportion of the actors change their decision at once.

Model 1b allows for a continuous distribution of factions. Because there is no noise in the system, the power ordering of factions cannot change. Therefore a single decision boundary between cooperation and defection will always exist, which may remain constant over time (during a cooperation or a defection phase) or move rapidly (during a cascade). This model takes the form of a Partial Differential Equation.

Parameter	Explanation
N	Number of factions
R_i^0	Raw resource available to faction i ($R_i^0 = 1/N$ in the basic model).
w	Defection 'intensity' controlling resource penalty and power gain
μ	Increment of time.
ρ	Power gain during defection relative to w.

Table 1: Description of parameters in the basic model.

Table 1 describes the parameters of the model, which for convenience we reproduce here. The decision to defect $D_i(t) = 1$ if $\eta_i(t) > 0$ and $D_i(t) = 0$ otherwise, where:

$$\eta_i(t) = R_i(t|D_i = 1, \mathbf{D_{-i}}) - R_i(t|D_i = 0, \mathbf{D_{-i}}). \tag{S1}$$

Cooperators pool and redistribute resource, $R_i(t|D_i = 0) = R_C^0 P_i(t)/P_C(t)$ (where $R_C^0 = \sum_{j:D_j=0} R_j^0$ and $P_C(t) = \sum_{j:D_j=0} P_j(t)$), whereas defectors retain resource with a penalty, $R_i(t|D_i = 1) = R_i^0 - w$. Power changes according to

$$\begin{aligned} P_i(t+1|D_i = 0) &= P_i(t) + \Delta t\left[\mu R_i(t) - \mathcal{N}(t)\right] \\ P_i(t+1|D_i = 1) &= P_i(t) + \Delta t\left[\mu\left(R_i(t) + \rho w\right) - \mathcal{N}(t)\right], \end{aligned} \tag{S2}$$

where $\mathcal{N}$ is a normalising constant to ensure $\sum_i \Delta P_i(t) = 1$. By substituting the normalising factor $\mathcal{N}$ into Eq. S2, defining the number of cooperators $C = N - \sum_{i=1}^{N} D_i$, the basic model can be written:

$$\begin{aligned} \frac{\Delta P_i(t|D_i = 0)}{\Delta t} &= \mu\left[\frac{C}{N}\frac{P_i(t)}{S(t)} - \frac{1}{N} - \frac{N-C}{N}w(\rho - 1)\right] \\ \frac{\Delta P_i(t|D_i = 1)}{\Delta t} &= \mu\left[\frac{1}{N} + w(\rho-1) - \frac{1}{N} - \frac{N-C}{N}w(\rho - 1)\right] \\ &= \mu\frac{C}{N}w(\rho - 1) \end{aligned} \tag{S3}$$

where $S(t) = \sum_{i=1}^{N}(1 - D_i)P_i(t)$ is the power held by the cooperators. For convenience we label this as *Model 1*.

Model 1a: A continuous-time description of Model 1 can be obtained by taking the limit $\Delta t \to 0$, which we will call Model 1a. The continuous time version of the model is special, because the original power ordering can not change. This can be exploited to obtain several theoretical results which do not exactly hold in the discrete time model, although the general features (such as overall defection patterns) do still hold. We order factions by their power (from high to low); now the number of cooperators C is a defection threshold with $D_i = 0$ for $i \leq C$, $D_i = 1$ for $i > C$. Therefore $S(t) = \sum_{i=1}^{C} P_i(t)$ is the power held by the cooperators, and additionally we can define $S_x(t) = \sum_{i=1}^{x} P_i(t)$ as the power held by all factions up to x. The power model becomes:

$$\begin{aligned} \frac{dP_i(t|i \leq C)}{dt} &= \mu \left[\frac{C}{N} \frac{P_i(t)}{S(t)} - \frac{1}{N} - \frac{N-C}{N} w(\rho - 1) \right] \\ \frac{dP_i(t|i > C)}{dt} &= \mu \left[\frac{C}{N} \right] w(\rho - 1) \end{aligned} \tag{S4}$$

Model 1b: We will make use of a continuous distribution of factions, by taking the continuous limit in faction space $x \in (1, N)$, leading to Model 1b:

$$\begin{aligned} \frac{\partial P(x,t|x \leq C)}{\partial t} &= \mu \left[\frac{C}{N} \frac{P(x,t)}{S(x,t)} - \frac{1}{N} - \frac{N-C}{N} w(\rho - 1) \right] \\ \frac{\partial P(x,t|x > C)}{\partial t} &= \mu \frac{C}{N} w(\rho - 1) \end{aligned} \tag{S5}$$

where we have defined $S(x,t) = \int_0^x P(x,t)dx$. We force $C \geq 1$ to preserve the special behaviour of the leading faction (now represented continuously by $x < 1$) who always cooperates. Expressed like this the model deals with infinitesimal densities. Since $S(x,t)$ appears on the right hand side, it will be convenient to work with this directly and recover faction power using $P(x,t) = \partial S(x,t)/\partial x$. We will write $S(x|t)$ when considering a fixed t and $S(t|x)$ for a fixed x; these equations are follow ODEs rather than PDEs.

Because of the dependence on the defection threshold C (which is defined in terms of the power distribution) searching for a general solution to the ensuing PDE in Model 1b is ambitious. However, we will be able to provide bounds to the distribution $S(x,t)$ that can lead to, and recover from, collapse into a defection state. From this we can bound the time taken for defection periods.

We are interested in the behaviour of all 3 models and will use results from each in the calculations. However, the focus is on properties of Models 1a-b that are also present in the simulations of Model 1.

S1.1 Behaviour when all factions cooperate

When all factions cooperate, the dynamics of Model 1a are simple. Substituting $D = 0$ into Equation S4 gives $S(t) = 1$ and hence:

$$\frac{dP_i(t)}{dt} = \mu \left[P_i(t) - \frac{1}{N} \right] \tag{S6}$$

which can be solved by separation of variables, with initial condition $P_i(t = 0) = P_i^0$ to give:

$$P_i(t) = \frac{1}{N} + \left(P_i^0 - \frac{1}{N} \right) \exp(\mu t) \tag{S7}$$

i.e. exponential departure from the initial deviation away from the mean initial resource (which was $1/N$). We can therefore solve for t_i^D, the time until faction i defects. This occurs when $R_i(t) = 1/N - w$, and as additionally we have $P_i(t) = R_i(t)$, we obtain:

$$t_i^D = \frac{1}{\mu} \log \left(\frac{w}{P_i^0 - 1/N} \right) \tag{S8}$$

Only the minimum time (hence $\arg\min_i(P_i^0) = P_N^0$) is relevant to the time of the first defection event.

S1.2 Defection cascades

A defection cascade is defined by defections occurring as a direct result of other defections. Faction N is first to defect and has $P_N(t) = R_N(t) = 1/N - w$; subsequent defectors have greater power.

Defecting factions have resource $R_i(t|D_i = 1) = 1/N - w$, hence the decision for faction C to defect requires:

$$\frac{C}{N} \frac{P_C(t)}{\sum_{j=1}^{C} P_j(t)} - \frac{1}{N} + p_w < 0 \tag{S9}$$

which can be solved for the lower bound $P_C(t) < b_C$ for the faction C to defect (recalling that faction $C = 1$ cannot defect):

$$b_C = \frac{1 - wN}{(C-1) + wN} \sum_{j=1}^{C-1} P_j(t) \tag{S10}$$

Note that $b_{C-1} \geq b_C$ since P_j is not decreasing with j.

We now work with the cumulative power $S_i(t) = \sum_{j=1}^{i} P_j(t)$, and solve for the case $b_C = P_C(t) = \Delta_i S_i(t)$ using the notation $S_i^*(t)$ for the cumulative power up to the boundary. Letting $P_C(t) = b_C$ acts as an effective upper bound for defection of faction C, since it places the minimum possible power with the remaining cooperators, making defection for one of them harder.

Recall that cooperators have small i, so S^* is the power in the remaining cooperators; defection of faction i will occur if the faction is too weak to remain with the other cooperators, i.e. $S_i^*(t)$ is too large. Rearranging Equation S9 for the boundary:

$$S_{i+1}^*(t) - S_i^*(t) = \frac{1 - wN}{i + wN} S_i^*(t) \tag{S11}$$

which when considered in Model 1b leads to

$$\frac{dS^*(x|t)}{dx} = \frac{1 - wN}{x + wN} S^*(x|t). \tag{S12}$$

Note that this restricts $0 < w < 1/N$. Equation S12 can be solved by separation of variables (recalling that $S(x = N|t) = 1$):

$$S^*(x|t) = \left(\frac{x/N + w}{1 + w} \right)^{1 - wN}. \tag{S13}$$

Instead of differentiating $S^*(x|t)$, which would approximate $S_i^*(t)$ via a piecewise linear function, we will obtain the best match with Models 1 and 1a if we compute the boundary for specific factions using differences of the cumulative power of factions:

$$P_i^*(t) = S_i^*(t) - S_{i-1}^*(t) = \left(\frac{i/N + w}{1+w}\right)^{1-wN} - \left(\frac{(i-1)/N + w}{1+w}\right)^{1-wN} \tag{S14}$$

For a defection cascade to occur in Model 1b, $S(x|t) > S^*(x,t)$ is a strict requirement on the distribution of power – any faction reducing the total cooperation power below this has sufficient power to continue cooperating. A defection cascade will stop at the first faction for which the bound does not hold. However, it is possible (and occurs in practice) that defection cascades instantaneously reach only a large proportion of the factions. The remaining few factions have power above the threshold, but their power continues to reduce, so more may join the defection. If N is large, several factions may avoid defection completely.

Defection cascades can occur instantaneously in Models 1a and 1b. For Model 1, assuming μ is small, a defection cascade changes the resource levels by a much larger amount than it would change under normal time evolution of the model. Therefore cascades in Model 1 (and Model 1a) take a very similar form, differing only qualitatively.

S1.3 Duration of epochs in the cycle

Collapse (and recovery) events are triggered by the whole cumulative power distribution falling above (below) the decision threshold. This is a complex procedure in general as smaller defection and cooperation events affecting a few factions can occur, which can change the shape of the resulting distribution.

We can obtain a bound on the maximum duration of cooperation and defection period. We assume that all factions act at the same time, do not defect independently during the cooperation phase, and do not cooperate independently during the defection phase. This implies that all defecting factions have the same power, as they experience an identical increase in power during the defection period, and therefore must experience the same decrease during the cooperation period.

This model maximises the time spent in a defection by restricting the first cooperation event. Consequently, the leading faction has been reduced to the minimum power possible and so the cooperation period starts from the fairest distribution of power possible. This maximises the time spent in the cooperation phase.

Collapse occurs when faction N defects, and recovery occurs when faction N can resume cooperation (as $P_i(t) = P_N(t)$ for all $i \geq 2$). As $(N-1)P_N(t) + P_1(t) = 1$, we can describe the dynamics in terms of a single quantity $P_N(t)$. Let τ_c be the time for collapse, τ_r be the time for recovery. Let $P_N(\tau_c) = P_N^c$ be the power of faction N at the collapse event, and $P_N(\tau_r) = P_N^r$ be the power at the recovery event. The duration of the cooperation phase is $t_a = \tau_c$ and the duration of the defection phase is $t_b = \tau_r - \tau_c$.

The condition for defection gives $P_N^c = 1/N - w$, and the condition for recovery (from Equation S10) gives $P_N^r = \frac{1-wN}{1+wN} P_1^r$ which (since power sums to 1) implies $P_N^r =$

$\frac{1}{N}\frac{1-wN}{1-w(N-2)}$. During the cooperation phase (from time 0 to t_c, duration t_a)

$$\begin{aligned}\frac{dP_i(t|C=N)}{dt} &= \mu\left(P_i(t)-\frac{1}{N}\right)\\ \implies P_N^c &= \frac{1}{N}+\left(P_N^r-\frac{1}{N}\right)\exp(\mu t_a)\end{aligned} \tag{S15}$$

leading to

$$t_a=\frac{1}{\mu}\log\left[N\left(w+\frac{1-Nw}{2}\right)\right]. \tag{S16}$$

Similarly, during the defection phase (from time t_c to t_r, duration t_b)

$$\begin{aligned}\frac{dP_N(t|C=1)}{dt} &= \mu\frac{1}{N}w(\rho-1)\\ \implies P_N^r &= P_N^c+t_b\mu\frac{1}{N}w(\rho-1)\end{aligned} \tag{S17}$$

leading to

$$t_b=\frac{1}{\mu(\rho-1)}\frac{(N-2)(1-Nw)}{1-w(N-2)}. \tag{S18}$$

This bound is extremely accurate when initial conditions are close to the assumptions (i.e. factions $2-N$ have equal power). Under some parameter and initial conditions, the distribution of power tends towards this structure, in which case the bounds are moderately close (but not exact due to intrinsic noise). If factions have a distribution broad distribution of power in stationarity, then the bound is poor. A significant factor is that defection and cooperation cascades do not occur instantaneously, and if multiple factions retain power above $1/N$ then the cooperation cascade is structured differently.

S1.4 Informative reformulations

When all $R_i^0 = R^0$ the resource defection penalty can trivially be written as a relative reduction in resource, rather than a subtraction:

$$R_i(t|D_i=1)=R^0-w=aR^0 \tag{S19}$$

where $a=1-w/R^0$. Similarly, the political gain for rebellion can be rewritten as a relative rather than absolute gain:

$$P_i(t+1|D_i=1)=P_i(t)+\Delta(t)\frac{\mu}{N}[R_i(t)+\rho w]=P_i(t)+\Delta(t)\frac{\mu\lambda}{N}R_i(t) \tag{S20}$$

where $\lambda=1+\rho w/(R^0-w)$. This form makes it clear that power under both choices are exponentially changing with different rates. The model has not changed under these reformulations.

S2 Model Extensions

S2.1 Unequal resource distribution

Most of the theory we have developed applies only to the case where all factions have equal access to resources. When these differ, each will have a different decision boundary and obey more complex dynamics (as e.g. when cooperating they will be diverging from different points). Although the mathematical details do differ, the intuitive reasoning remains the same: each faction has a decision boundary, and changes of decision will still have a cumulative effect on other factions' decision boundaries. Therefore (provided all factions can actually afford to defect) the qualitative dynamics remain the same.

In Figure 3e we use a parametric model for the unfairness of the resource distribution, parameterised by the inequality parameter κ:

$$R_j^0 = \frac{\exp(\kappa j)}{\sum_{i=1}^{N} \exp(\kappa i)} \tag{S21}$$

Figure S1 shows the structure of this model as the intrinsic resource available to factions, and the initial conditions, are varied. When the initial distribution of power is correlated with resource (Figure S1A), then the dynamics of the model are almost unchanged from the uniform resource model. However, political leadership can be taken from the resource-weak by a resource-strong faction (Figure S1B). It is possible for an faction to remain leader when they only have a moderate resource (Figure S1C).

Since the most powerful faction is $j = 1$ and the most resource-rich faction for $\kappa > 0$ is $j = N$, there is a conflict between power and raw resource. Hence Figure 3e involves a reversal of the capital for a wide range of κ. However, for smaller κ the reversal does not occur and a resource-poor faction remains in power. Figure S1 illustrates how this results in *lowered* overall conflict rate, because average inequality remains at a lower level. When resource rich factions are in power (Figure S1AB, other factions much defect frequently to maintain their position. When resource poor factions are in power (Figure S1c), resource-rich factions do not need to defect much, and other resource-poor factions are unaffected, resulting in a lower defection rate.

S2.2 Uncertain outcomes

Power has been modelled using deterministic dynamics conditional on the defection choices. We relax this unrealistic assumption by allowing noise in the outcome of the political power process. If we let $\Delta t P_i^D(t)$ be the deterministic prediction for the change in power from Equation S2, then the power follows

$$P_i(t) = \Delta t P_i^D(t) + \Delta t N(0, \sigma_p), \tag{S22}$$

which in the continuous time limit (cf Model 1a) can be seen as an Ornstein-Uhlenbeck (OU) process. This process is 'mean reverting' to the deterministic solution but does deviate on average by $\sigma_p/2\mu$ (by rescaling and taking the continuous limit).

The appropriate scale for the noise is the difference between the power of the factions. From Section S1 this is $O(1/N)$ so $\sigma_p/2\mu \ll 1/N$ for the deterministic dynamics to dominate. Figure 3f and Figure S2 support this. Noting that we used $\mu = 0.05$ and $N = 11$ here leading to $\sigma_p \ll 0.009$, the asymptotics have worked surprisingly well. Increasing

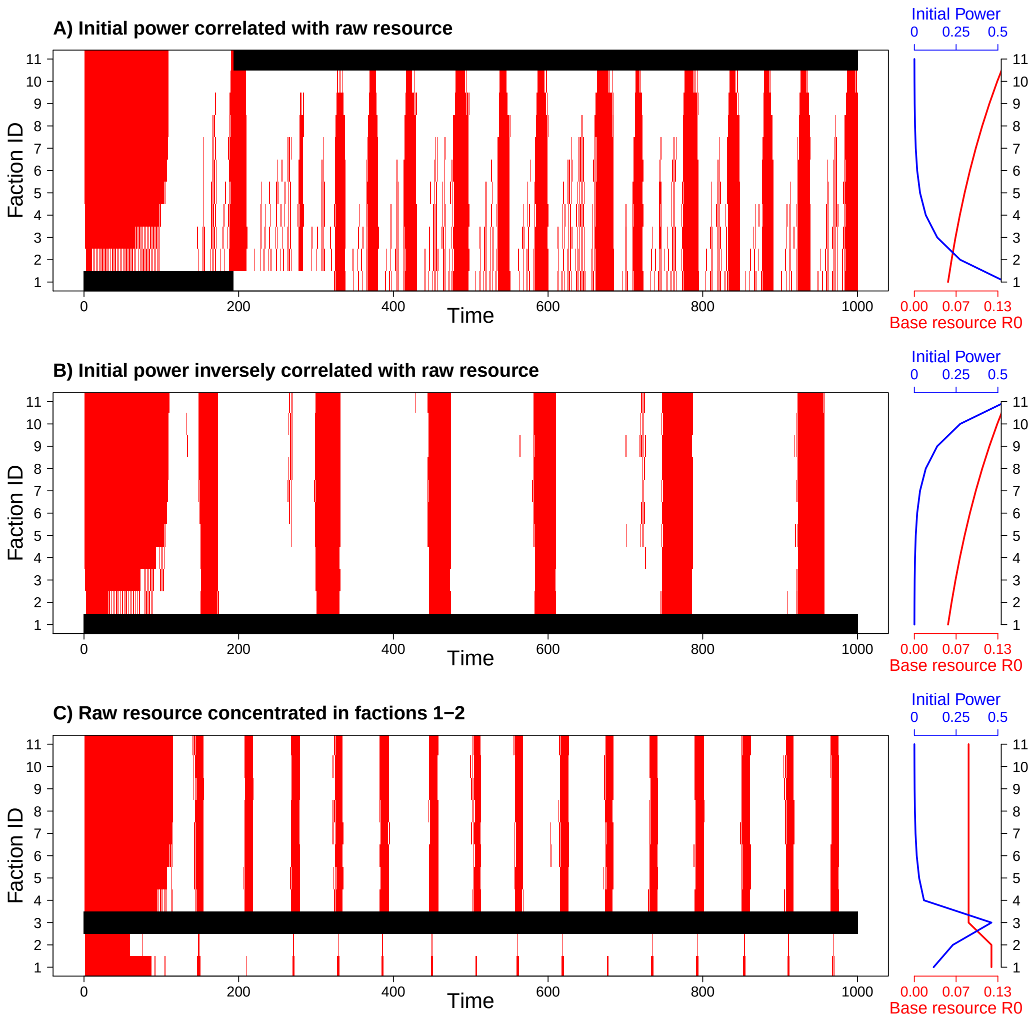

Figure S1: Effect of changing raw resource levels. The left plot shows the defection behaviour as in Figure 1C, and the right plot shows the initial power distribution (blue line, top axis) and base resource level R_i^0 (red line, bottom axis). Shown are A) $\tau = 0.1$, B) $\tau = -0.1$, and C) initial power is distributed such that the highest resource factions are not leaders.

σ_p, leader replacement occurs first, then periodicity is lost, followed by infrequent state formation and finally a failure to coordinate collapse events.

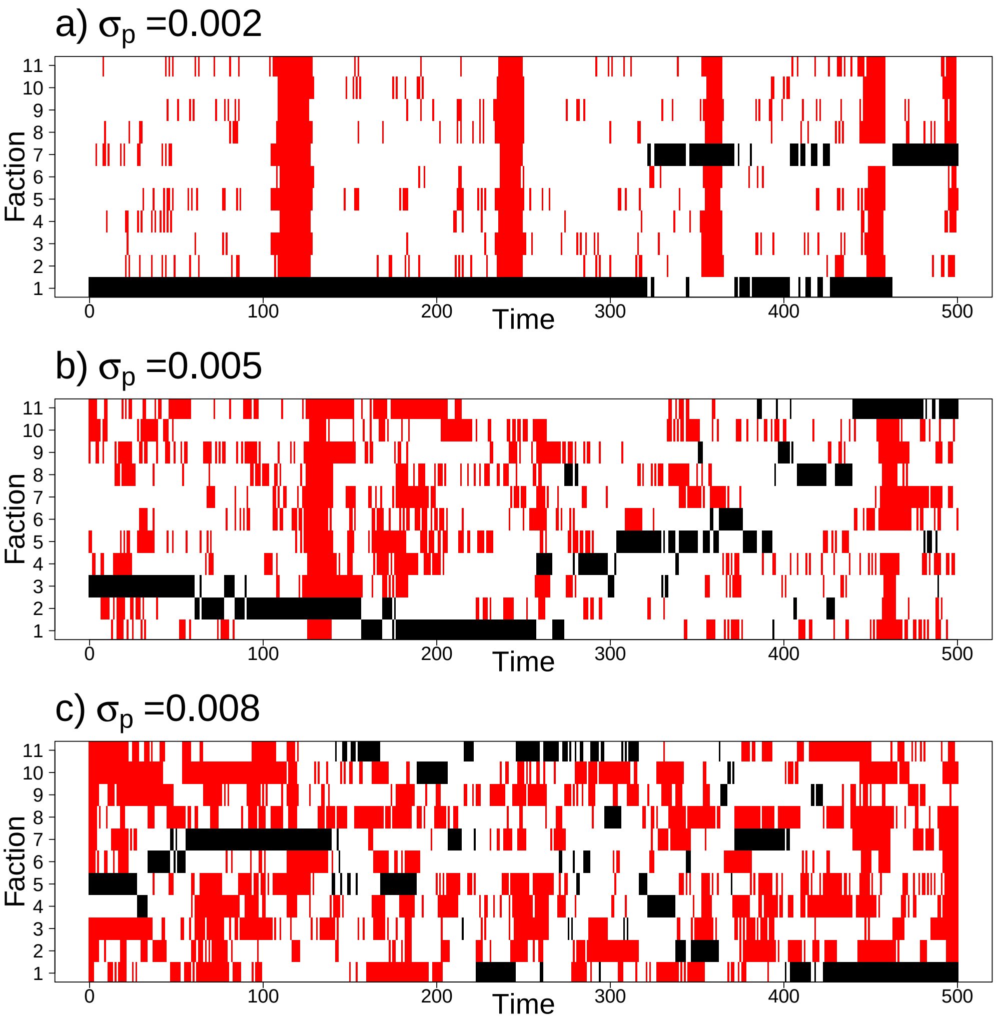

Figure S2: Effect of changing the randomness in power outcomes σ_p. a) $\sigma_p = 0.002$, for which leader replacement is rare but possible. b) $\sigma_p = 0.005$, for which periodicity has been broken down but collapse events persist. c) $\sigma_p = 0.008$ for which noise is significant and state formation is rare (but possible), and collapse events are poorly coordinated.

S2.3 Biased decision making and random choices

To generate an 'imperfect decisoon model' we replace the short-term resource optimising decision rule for η with a random function taking the form

$$\eta_i(t;\tau,\sigma,\beta) = R_i(t|D_i = 1) - R_i(t|D_i = 0) + \beta[2D_i(t-1) - 1] + \sigma G(t;\tau), \tag{S23}$$

where β is the bias towards the previous choice. $G \sim \mathrm{GP}(\tau)$ is a Gaussian Process, which is a random function with expected mean 0 and standard deviation 1. Specifically, for a specified set of times $t_1 \cdots t_N$, we define $G(t_1, \cdots, t_N) \sim \mathrm{MVN}(0, \mathrm{K})$ i.e. multivariate normally distributed with covariance matrix $K_{i,j} = \tau^2 |t_i - t_j|^2$. One of the reasons to use a Gaussian Process is that they defined for all times but only need to be evaluated at the times where they are needed.

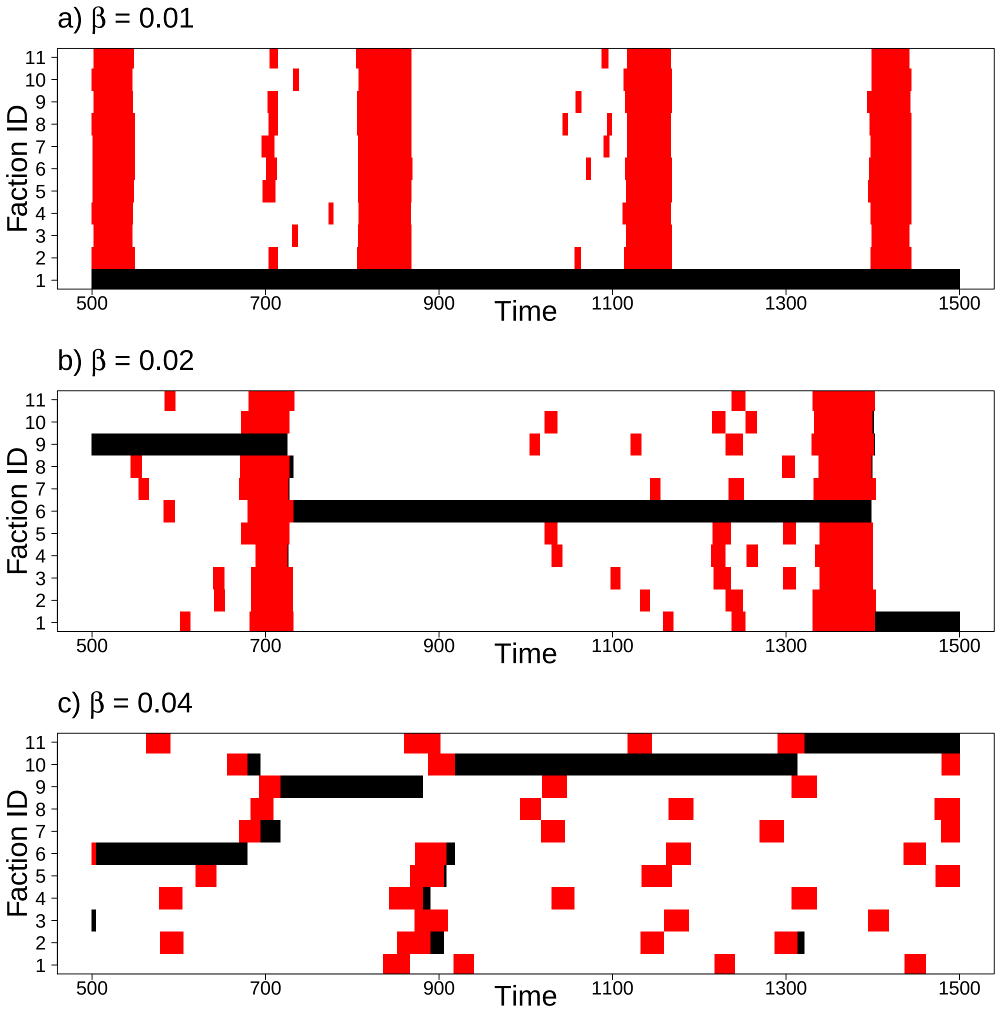

Figure S3: Behaviour of the Imperfect decision model with persistent choices, varying β. a) Small $\beta = 0.01$ produces behaviour that is qualitatively the same as $\beta = 0$, with a slightly longer period. b) Intermediate $\beta = 0.02$ allows leader turnover and increases the period further. c) Large $\beta = 0.04$ prevents full scale collapse events but increases the rate of collapse events, increasing the leader turnover rate. As before, $w = 0.02$, $\rho = 20$,$\mu = 0.01$ and $N = 11$.

Figure S3 illustrates the effect that a tendency to keep the same decision (parameterised by β) has on the behaviour of the model. In general, it produces longer periods of both defection and cooperation, as factions are reluctant to change. However, this has the effect of forcing a more fair initial distribution of power in the forming state, which permits the state to survive for still longer. Defections for a single faction have a defined minimum length, since it takes a significant improvement of power to reverse a decision. When β is moderate (Figure S3b), repeated defection can completely erode the leaders resource benefit, resulting in leader turnover. For very large β (Figure S3c) the behaviour of others does not affect choices very strongly compared to the desire to maintain the previous action. In this case defection cascades are partially prevented.

The model with $\tau = 0$ has independent Gaussian noise which is faster to simulate from. The tendency not to change decisions can be easily be motivated from a psychological perspective, or using internal politics if factions are groups of individuals. For example, a faction leader may make a promise to cooperate or defect, which is difficult to reverse without losing face. It can also be seen as risk aversion for the unknown effects of new actions – factions may find it difficult to predict whether others will defect if they do.

Figure S4 demonstrates how the model breaks down in the presence of noise. Small σ relative to the intrinsic power variability for non-leading factions has little effect on the dynamics. However, as σ increases it becomes more likely that factions will defect during stable cooperation. Moderate σ leads to a reduction in the length of collapse periods (Figure S4b). High σ lets factions make choices 'randomly' (Figure S4c), which prevents the possibility of state formation as no faction can remain in power.

However, correlated decisions can potentially permit cooperation in the presence of very large decision noise. Figure S5a-b shows the behaviour in the intermediate noise case with $\sigma = 0.012$. With $\tau = 5$ (Figure S5a) cooperation periods have markedly increased average cooperation levels from the $\tau = 0$ case (Figure S4b), cooperation periods are shorter and collapse times are less predictable. Increasing τ to 25 (Figure S5b) restores the very clear distinction between cooperation and defection periods. This persists even at high σ (Figure S5c), with the effect that leader turnover is now possible as factions may refuse to cooperate for long enough to remove the leaders natural resource advantage.

The reason that highly correlated noise can support cooperation and defection periods in our model is that each faction has a new equilibrium resource level that they will change their action at (in the short term). If this changes more slowly than the internal political dynamics, then they will change behaviour due to the effect of others rather than due to their own random choices.

S2.4 Spatial structure

Some political scenarios are best described with a spatial model. For example, factions may be local leaders of villages, or semi-autonomous regions of a larger state. Spatial structure can be introduced via the relative advantage that cooperation confers. It is natural that both the resource penalty for defection, and the political gains from doing so, should depend on physical location. We can achieve this by replacing w by w_i, which is a function of distance from the political leader. Specifically, we allow an exponential decay in the impacts of defection with distance:

$$w_i = w^* \exp\left(-|x_i - x_C| w_d / N\right) \tag{S24}$$

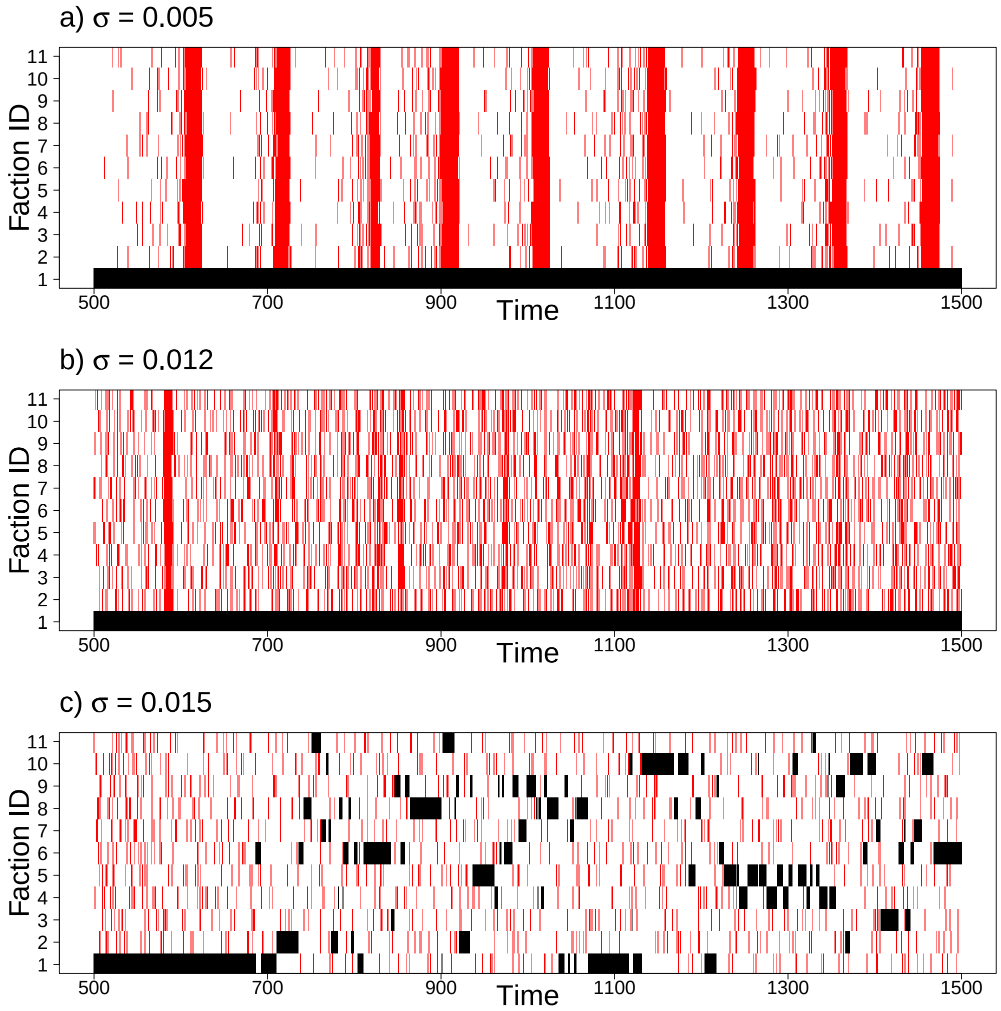

Figure S4: Behaviour of the imperfect decision model with random choices, varying σ but fixed $\tau = 0$. In a) low noise levels lead to the same qualitative dynamics as in the no-noise case, in b) medium noise levels reduce coordination of collapse, leading to less define collapse events. In c) high noise levels prevent the construction of a permanent 'state', with turnover of the leading faction possible but state formation involving all factions is not possible. As before, $w = 0.02$, $\rho = 20$,$\mu = 0.01$ and $N = 11$.

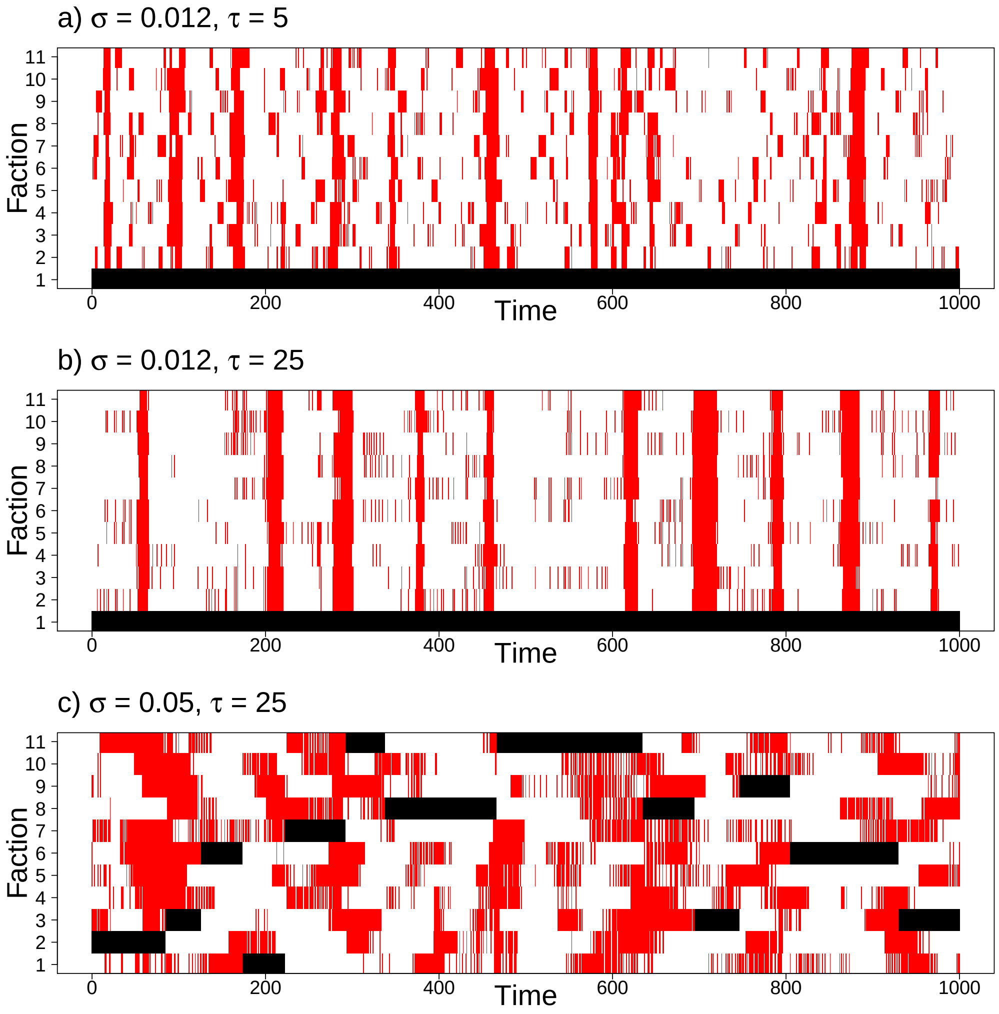

Figure S5: Behaviour of the imperfect decision model with random choices, varying σ and decision correlation time $\tau \neq 0$. a) and b) have σ comparable to Figure S4b,but vary the decision correlation time τ; c) is comparable with Figure S4c with large τ. As before, $w = 0.02$, $\rho = 20$,$\mu = 0.01$ and $N = 11$.

where w^* is a normalising factor to ensure the average effect is the same as the non-spatial model, $E(w_i) = w$, and w_d is the spatial decay rate. When $w_d = 0$ the model reduces to the basic model. The factor N is included to account for the size of the system, as $x_i = i$ is the spatial location of each faction (we consider them on a ring so that faction 1 is next to faction N, to remove boundary effects).

The spatial model in Figure S6 allows for a variety of different scenarios. Interpreting cooperation as a political state, this will grow from the ‘capital’ (the leading faction) and will collapse as in the non-spatial model. Collapse may be from the outside-in (Figure S6b)

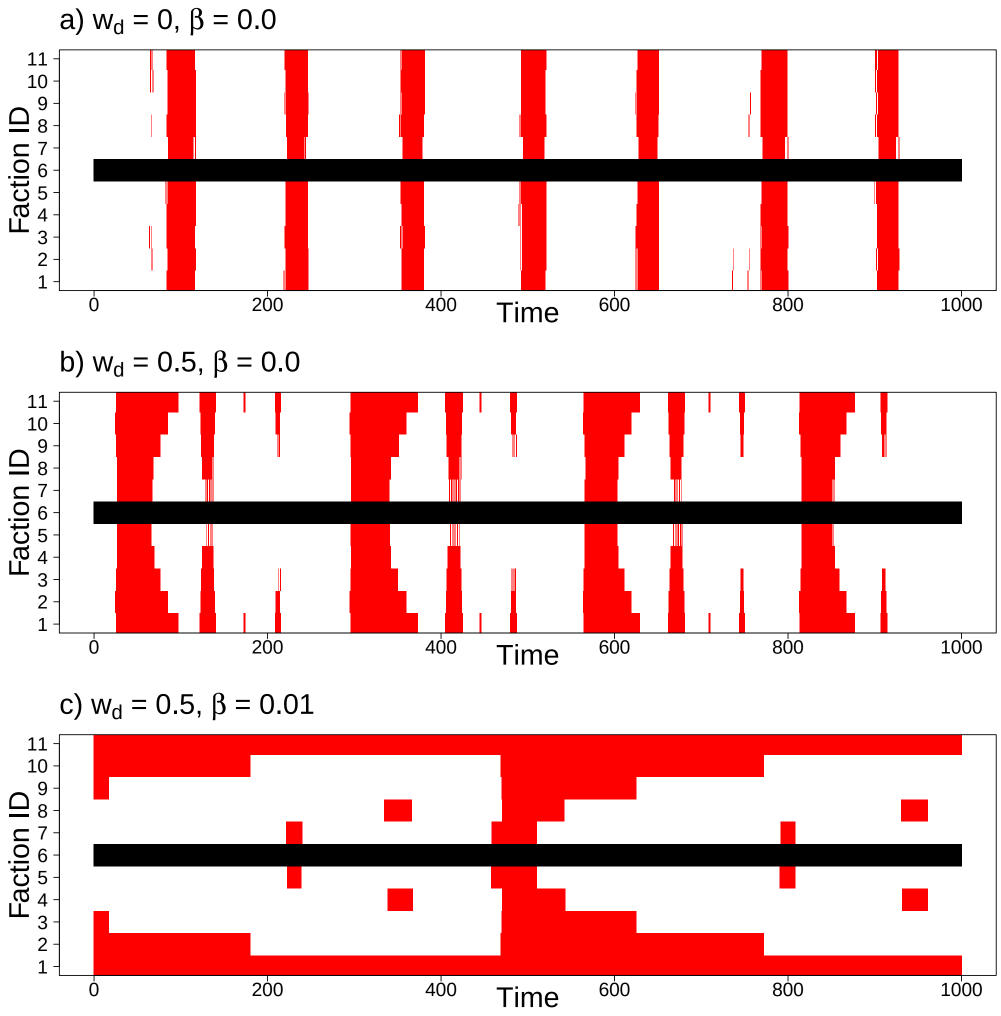

Figure S6: Behaviour of the spatial model (Model 3) with different values of the spatial structure strength w_d. a) $w_d = 0$ and $\beta = 0$, for comparison. b) $w_d = 0.5$ and $\beta = 0$. c) $w_d = 0.5$ and $\beta = 0.01$. As before, $w = 0.02$, $\rho = 20$ and $N = 11$, but we use $\mu = 0.005$ for greater temporal resolution of the collapse events.

or the inside-out (Figure S6c). There may be a well defined maximum spatial extent. As in the non-spatial model, the leader will remain stationary unless the tendency to repeat the last action (β) is high enough. Because the change in power is highly dependent on location, the duration of cooperation and defection periods changes drastically, but the qualitative features of the non-spatial model remain.

S2.5 Modified intrinsic noise

We chose to define the basic model as an iterated game. However, this has consequences for the way that noise enters the system. There is nothing stochastic in the definition of the model, but the discretisation of time can produce 'chaotic' dynamics as small variations in the value of political power have large effects if they interact with the decision boundary between defection and cooperation. Additionally, power changes relatively rapidly during defection, which means that non-linear effects of the interaction of various states will make the continuous time version of the model behave differently.

Intrinsic noise arises from the discrete nature of the choices and the times at which those are made. If the power of state i changes by $\delta P_i(t)$ and it has a decision boundary at P_i^* then there are a range of power values that lead to the same outcome of a change in decision. Since power changes non-linearly, the particular values of power can get out of phase, but can also be reset into phase during coordinated defection by changing the order of cooperation. This can lead to semi-regular noise structures, such as that observed in Figure 1. From Equation S3 the noise is of intrinsic magnitude $O(\mu/N)$ (with situation-specific dependence on the other parameters).

There are three possible views of intrinsic noise:

1. Treat the underlying continuous-time, noise-free model as the model of interest (Model 1a, described by Equation S3). Intrinsic noise is viewed as a numerical integration error, and alternative numerical schemes could be considered.

2. Treat μ as a real parameter in the model, and treat the chaotic dynamics as a 'real' source of noise.

3. Consider alternative models that characterise the discretisation differently, and focus on properties of the system that are common to all models.

Although it would be interesting to study, we have not performed numerical integration. This is because a) a somewhat specialist approach would be required to handle the choice dynamics, and b) we do not believe that the noiseless version of the model is 'closer to reality' than the discretised versions. We also don't treat μ as an important parameter, again because we believe that extrinsic noise will be present in real-world examples. We instead focus on features that are robust to the level and type of intrinsic noise. To this end we construct an additional variation of the basic model.

Model 1c: rather than updating *all* factions at every timestep, we increment time by $\delta t \sim \exp(1/N)$ (so that $E(\delta t) = 1/N$) and update a single, *random* faction. We note that this takes the form of the Gillespie Algorithm and is appropriate if factions are independently updating their decisions at rate 1. (Since the number of factions is constant in our model, there is no interesting difference between this model and that with $\delta t = 1/N$ exactly). Time increments by 1 on average after N iterations of the new timestep:

1. A random faction i can choose whether to cooperate (determining $D_i(t)$ from $\eta_i(t)$). All other factions perform their previous action.

2. The actual resource obtained is evaluated.

3. The corresponding power changes are computed with Equation S2.

This is slightly preferable to the original definition as we have removed one cause of chaotic dynamics (the simultaneous decision problem). This prevents 'alternating' where two sets of factions are exactly out of phase with one another (seen in Figure 1a).However, this algorithm takes $O(N)$ more computing power, contains an explicit form of noise, and does not produce any qualitative difference in behaviour.

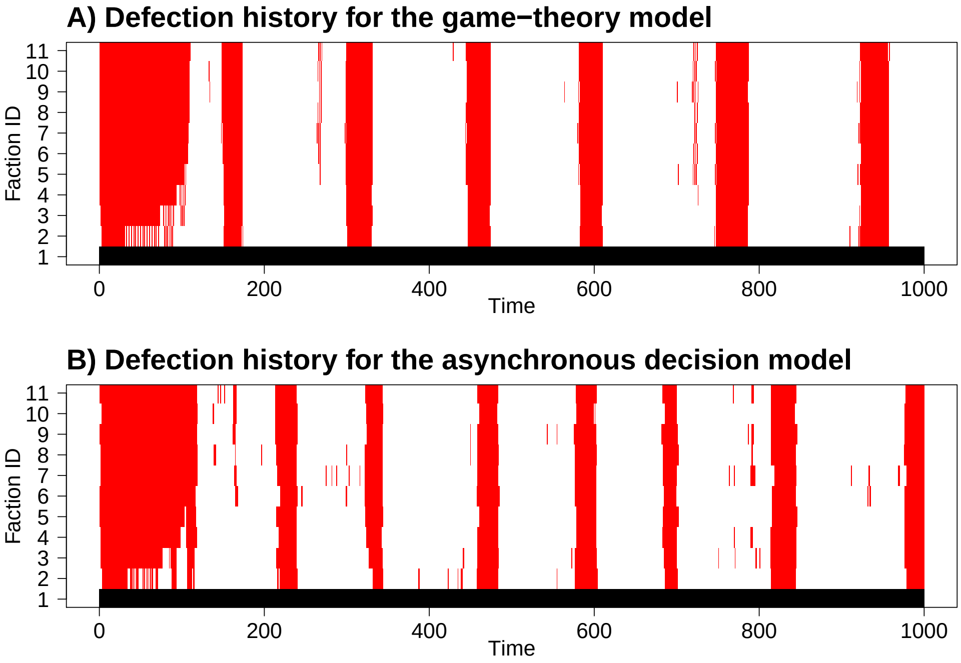

Figure S7: The modified intrinsic noise model, comparing a) the standard synchronised decision model, and b) the asynchronous decision model. Asynchronous decisions do not change the qualitative periodic dynamics, but the period has changed slightly.

Figure S7 shows both the synchronous and asynchronous models, which display the same overall features of periodicity formed by collapse/recovery cycles. The nature of the intrinsic noise has changed; although the discretisation noise is $O(N)$ smaller, there is additional noise in the random decision order. This leads to a change how defection occurs in the cooperation phase - it is more common, less coordinated, and (unlike the synchronous case) does not involve the same ordering of factions. A consequence is that both defection periods and cooperation periods are slightly shorter.

There is not a trivial reparameterisation that leads to the same periodicity. However, a parameter mapping exists to retain the same periodic structure, and the models will only be distinguishable by their fine-scale structure.

S2.6 Non-uniform defection penalty

We might believe that the magnitude of the penalty for defection should depend on the number of cooperators, such that few defectors get a larger relative resource penalty than

when there are many defectors. This would also eliminate the somewhat artificial cooperation benefit enjoyed by the political leader even when there are no other cooperators. Most reasonable models will make it easier for full cooperation and defection cycles to occur. This is because defecting is more difficult when there are few defectors, and cooperating is more difficult when there are few cooperators. Provided that decision changes are still possible for some achievable political power, then cycles will still occur and the qualitative dynamics will be as the basic model. Hence we have focussed on the uniform defection penalty model as it is the 'hardest' model we considered for producing collapse events.

We substitute a variable resource penalty for w into the basic model:

$$w_i(t|S_i = 1, \{S_j\}) = \frac{Nw}{N-2}\left[\left(\sum_{j;S_j=0} R_j^0\right) - R_i^0\right] = w\frac{N-D-1}{N-2}. \qquad \text{(S25)}$$

Here, when 'all defect' (except the leader) $D = N - 1$ the penalty is $w_i = 0$; when all cooperate, the penalty for the first defection (with $D = 1$) is $w_i = w$.

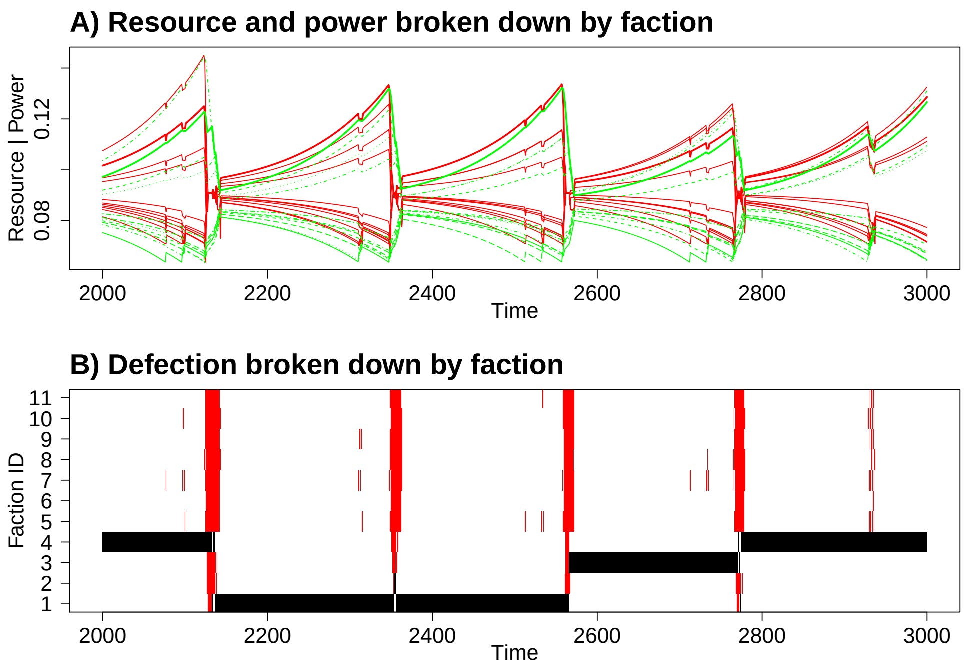

Figure S8: Plot of the dynamics in the variable resource penalty model, with penalty for defection proportional to the number of cooperators. A) shows the resource (red) and power (green), whereas B) shows the defection history.

Figure S8 shows the behaviour of this model, which allows for leader turnover in a non-trivial way. In simulations, we find that the first few factions (in initial political power) all manage to lead cooperation phases, but that politically poor initial circumstances prevent

ever becoming the leader. This dynamic occurs because the decision boundary during defection phases has moved significantly higher. A defector considering joining a single cooperator has a boundary with w replaced by $w/(N-2)$, and therefore the boundary is an order of N closer to the leader's. Intrinsic or extrinsic noise is much more likely to lead to a replacement event. However, only factions who retain above-average power can participate in this lead, as others are marginalised during the cooperation phase.

S2.7 Non-linear relationship between power and resource

Power changes might not scale linearly with resource. We can consider a modification of Equation S2 in which power changes at some power of R_i (which can depend on the defection status):

$$\begin{aligned} P_i(t+1|D_i=0) &= P_i(t) + (\mu/N)R_i(t)^{\alpha} - \mathcal{N}(t) \\ P_i(t+1|D_i=1) &= P_i(t) + (\mu/N)[R_i(t)+\rho w]^{\beta} - \mathcal{N}(t) \end{aligned} \tag{S26}$$

where α and β take values in $(0,\infty)$ and $\mathcal{N}(t)$ is redefined to maintain a total power of 1. A trivial examination of the continuous time limit of this model (as Model 1a) makes it clear that this simply affects the rate at which power exponentially departs from $1/N$ and therefore cannot have an important consequence for the modelling.

An additional model that might be considered allows resource to be non-linear in power within the cooperators. This again cannot have an important impact as it only changes the decision boundary, and can otherwise be written in terms of Model 1b with $\beta = 1$ and $\alpha \neq 1$.
